# Triglyceride-glycated hemoglobin index as a superior predictor of type 2 diabetes risk in a large-scale retrospective cohort study

**DOI:** 10.1038/s41598-025-05786-4

**Published:** 2025-07-01

**Authors:** Changchun Cao, Yibing Zan, Yong Han, Haofei Hu, Jianjun Long, Yulong Wang

**Affiliations:** 1 Department of Rehabilitation, Longgang E.N.T Hospital & Shenzhen Key Laboratory of E.N.T, Institute of Ear Nose Throat (E.N.T), Shenzhen, 518000 Guangdong Province China; 2 Department of Rehabilitation, Shenzhen Dapeng New District Nan’ao People’s Hospital, Shenzhen Second People’s Hospital, Shenzhen, 518000 Guangdong Province China; 3 Department of Emergency, Shenzhen Second People’s Hospital, The First Affiliated Hospital of Shenzhen University, Shenzhen, 518000 Guangdong Province China; 4 Department of Nephrology, Shenzhen Second People’s Hospital, The First Affiliated Hospital of Shenzhen University, No.3002, Sungang West Road, Futian District, Shenzhen, 518000 Guangdong Province China; 5 Department of Rehabilitation, Shenzhen Second People’s Hospital, The First Affiliated Hospital of Shenzhen University, No.3002, Sungang West Road, Futian District, Shenzhen, 518000 Guangdong Province China

**Keywords:** Triglyceride-glycated hemoglobin index, Type 2 diabetes, Triglyceride, Glycated hemoglobin, Diseases, Endocrinology

## Abstract

Early detection of individuals at elevated risk for type 2 diabetes (T2D) is critical for effective prevention strategies. We developed a novel metabolic marker, the triglyceride-glycated hemoglobin index (TyH-i), which integrates lipid and glycemic parameters to improve T2D risk prediction. This study sought to evaluate the relationship between TyH-i and T2D risk and to examine its predictive performance with the triglyceride-glucose index (TyG-i). A large-scale retrospective cohort study was conducted using data from 15,464 Japanese adults without T2D at baseline. The TyH-i was calculated as Ln[glycated hemoglobin index (%)×triglycerides (mg/dL)/2], and its association with incident T2D was assessed using Cox proportional hazards models and smooth curve fitting and cubic spline functions. The predictive performance of the TyH-i was compared with the TyG-i using receiver operating characteristic curves. Over a median follow-up of 5.39 years, 373 participants developed T2D. After accounting for confounders, the TyH-i was significantly associated with T2D risk (HR: 1.55, 95% CI: 1.22–1.97, P = 0.00031). Additionally, a J-shaped relationship between the TyH-i and incident T2D was identified. A significant positive correlation was identified between TyH-i and T2D only when TyH-i levels were above 4.92 (HR: 1.73, 95%CI: 1.35–2.23, P < 0.0001). Furthermore, TyH-i and TyG-i possess comparable discriminatory ability in predicting incident T2D (AUC: 0.751 vs. 0.750, P = 0.8873). Moreover, TyH-i yielded lower Akaike Information Criterion (AIC) and Bayesian Information Criterion (BIC) values than TyG-i (AIC: 3211.60 vs. 3211.91; BIC: 3226.89 vs. 3227.20), indicating a more parsimonious model with better overall model fit. The TyH-i is a novel and effective predictor of T2D risk, exhibiting a non-linear relationship with T2D. Furthermore, TyH-i and TyG-i exhibit comparable discriminatory abilities in predicting incident T2D. These results highlight the potential of TyH-i as a valuable instrument for T2D risk stratification, particularly in individuals with elevated TyH-i levels.

**Supplementary Information:**

The online version contains supplementary material available at 10.1038/s41598-025-05786-4.

## Introduction

Type 2 diabetes (T2D), a long-term metabolic disease marked by sustained hyperglycemia, has emerged as a global health epidemic. Over 500 million individuals aged 20–79 years, accounting for approximately 10.5% of this age group, were living with diabetes worldwide in 2021, based on the International Diabetes Federation^1^. The global prevalence of diabetes is projected to surpass 700 million by 2045^1^. T2D substantially increases the risk of a wide range of complications, such as heightened mortality, cardiovascular disease, diabetic retinopathy, poor stroke outcomes, and chronic kidney disease^2–5^. Consequently, the early detection of individuals at high risk for T2D is essential for implementing effective public health interventions and preventing disease progression.

In recent years, metabolic indices that integrate lipid and glycemic parameters have garnered significant attention as practical tools for predicting T2D risk. In particular, the triglyceride-glucose index (TyG-i) has been extensively studied and validated as both a marker of risk and a reliable predictor for insulin resistance and T2D^6–12^. However, the TyG-i has a notable limitation: it does not account for long-term glycemic control, which is a critical component of T2D pathophysiology. Glycated hemoglobin (HbA1c), a well-established marker reflecting average blood glucose concentrations over the past 2–3 months, offers a more comprehensive glycemic status assessment than fasting glucose alone^13^. Despite its clinical importance, few studies have investigated the integration of HbA1c into metabolic indices for T2D risk prediction.

To address this gap, we developed a novel metabolic marker, the triglyceride-glycated hemoglobin index (TyH-i), which combines triglyceride (TG) levels and HbA1c to capture better the interplay between lipid metabolism and long-term glycemic control. This study aimed to assess the correlation between TyH-i and T2D risk in a large Japanese cohort and to compare its predictive performance with that of the widely used TyG-i.

## Methods

### Data source and study participants

The present study analyzed data from the NAGALA database, a longitudinal cohort study conducted at Murakami Memorial Hospital in Gifu, Japan, over the period from 1994 to 2016. The dataset was obtained from the Dryad Digital Repository, and all analyses complied with the repository’s data usage policies. Murakami Memorial Hospital’s ethics committee approved the original research, and each participant provided written informed consent^14^. For the secondary analysis, no additional approval from the institutional review board was necessary. Additionally, our study was conducted in compliance with the principles of the Declaration of Helsinki, with all procedures carried out in accordance with applicable guidelines and regulations.

**Fig. 1:**
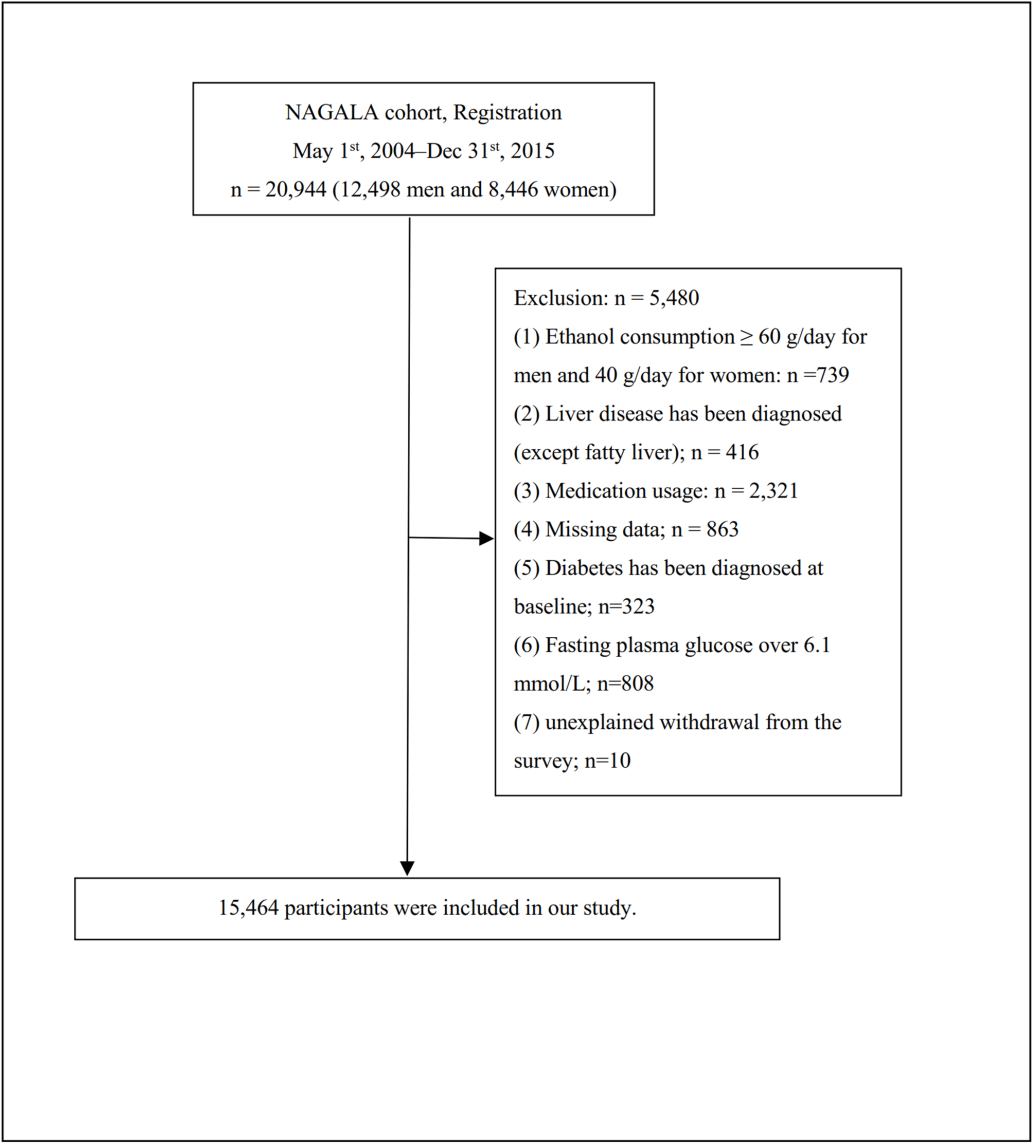
Study Population.

The study population included individuals who underwent at least two health checkups at Murakami Memorial Hospital^14^. Participants were excluded based on criteria from the original study, including pre-existing liver disease, excessive alcohol consumption at baseline, use of any medication, missing data, a confirmed diagnosis of diabetes at baseline, missing fasting plasma glucose (FPG) values, or unexplained withdrawal from the study. Of the initial 20,944 participants, 15,464 remained after applying the exclusion criteria (Fig. 1).

### Data collection

Baseline data were collected during routine health checkups and included demographic, clinical, and laboratory variables by trained staff. Covariates included sex, age, body mass index (BMI), systolic blood pressure (SBP), diastolic blood pressure (DBP), alcohol intake, smoking status, exercise habits, total cholesterol (TC), high-density lipoprotein cholesterol (HDL-C), aspartate aminotransferase (AST), alanine aminotransferase (ALT), gamma-glutamyl transferase (GGT), and FPG. Basic demographic information was collected through a structured questionnaire, while all blood examinations were performed using the MODULAR ANALYTICS system (Hitachi High-Technologies Corp. Ltd, Tokyo, Japan). The TyH-i was determined using the formula: TyH-i = Ln[HbA1c (%) × TG (mg/dL)/2]. Similarly, the TyG-i was determined using the formula: TyG-i = Ln[FPG (mg/dL) × TG (mg/dL)/2]^7^.

### Outcome measures

T2D was diagnosed based on self-reported medical history, HbA1c levels ≥ 6.5%, or FPG levels ≥ 7.0 mmol/L^15^.

### Statistical analysis

Baseline characteristics of the study population were summarized using descriptive statistics. Continuous data are reported as means ± standard deviations (SD) or medians with interquartile ranges (IQR), while categorical variables are reported as frequencies (%). Differences between groups were assessed using the chi-square test for categorical data, and the Kruskal-Wallis H test or one-way ANOVA for continuous data.

In adherence to the STROBE guidelines, three sequential multivariate Cox proportional hazard regression models were constructed to calculate hazard ratios (HR) and 95% confidence intervals (CI) for evaluating the effect of TyH-i levels on T2D. Model 1 was an unadjusted model with no covariates included. Model 2 adjusted for alcoholic intake, sex, exercise habits, age, BMI, smoking status, and SBP. Model 3 further expanded on Model 2 by incorporating additional adjustments for ALT, TC, AST, HDL-C, GGT, and FPG. Additionally, collinearity analysis identified DBP as collinear with other covariates, leading to its exclusion from the final multivariate Cox proportional hazards regression model (Table S1).

The robustness of the results was evaluated through sensitivity analyses. Considering the well-established associations between advanced age and obesity with T2D^16,17^, sensitivity analyses were performed by excluding participants aged ≥ 60 years or those with BMI ≥ 25 kg/m^2^ to assess the strength of the association between TyH-i and T2D risk.

This study employed Cox regression with smooth curve fitting and cubic spline functions to investigate the potential non-linear correlation between TyH-i and T2D. If non-linearity was detected, inflection points were identified using a recursive algorithm. This process involved dividing the dataset into two segments at an arbitrary starting point within the range of TyH-i values. Cox proportional hazards regression models were then applied separately to the left and right segments of the inflection point. The log-likelihood ratio test was used to determine the best-fitting model for assessing the association between TyH-i levels and T2D.

Further stratified analyses were conducted based on alcoholic intake, sex, exercise habits, age, BMI, and smoking status. Potential interactions among the stratified groups were evaluated using the likelihood ratio test.

The predictive performance of TyH-i was compared with the TyG-i using receiver operating characteristic (ROC) curve analysis, with the area under the curve (AUC) as the primary measure of discrimination. Model calibration was assessed using calibration plots generated by bootstrap resampling (1,000 iterations) and the Hosmer-Lemeshow goodness-of-fit (H-L) test. Model selection criteria, including Akaike Information Criterion (AIC) and Bayesian Information Criterion (BIC), were calculated for each model to quantify goodness of fit and parsimony. To further investigate the incremental predictive value of the TyH index over the TyG index, we calculated both the categorical and continuous Net Reclassification Improvement (NRI) and the Integrated Discrimination Improvement (IDI). Moreover, we used a cohort of 8304 Chinese participants from the China Health and Retirement Longitudinal Study (CHARLS) for the external validation.

All results were reported following the STROBE statement guidelines. Statistical computations were performed utilizing the Empower-Stats software Version 5.2 (www.empowerstats.net, X&Y solutions, Inc. Boston, Massachusetts). Statistical significance was defined as a two-sided P-value below the threshold of 0.05.

## Results

### Participant characteristics

A total of 15,464 participants without T2D at baseline were included in the present study, with a mean age of 43.71 ± 8.90 years, of whom 54.51% (*n* = 8,430) were male. Table 1 presents the baseline characteristics stratified by TyH-i quartiles. In the highest TyH-i quartile (Q4), participants demonstrated higher ALT, TC, DBP, TG, FPG, SBP, AST, BMI, alcohol intake, GGT, and TyG-i. In addition, this group exhibited lower levels of HDL-C, a higher proportion of males and smokers, and a lower proportion of individuals engaging in exercise habits.

**Table 1:** The characteristics of participants.

TyH-i	Q1 (≤−4.72)	Q2 (4.72 to ≤ 5.12)	Q3 (5.12 to ≤ 5.56)	Q4 (> 5.56)	*P*-value
Participants	3863	3847	3888	3866	
Sex					< 0.001
Female	2777 (71.89%)	2014 (52.35%)	1466 (37.71%)	777 (20.10%)	
Male	1086 (28.11%)	1833 (47.65%)	2422 (62.29%)	3089 (79.90%)	
Age(years)	40.50 ± 8.13	43.36 ± 8.82	45.03 ± 8.91	45.93 ± 8.73	< 0.001
Alcoholic intake (g/wk)	1 (0–22)	1 (0–60)	2.80 (0–84)	12 (1–108)	< 0.001
Smoking status					< 0.001
Never-smoker	2933 (75.93%)	2430 (63.17%)	2072 (53.29%)	1596 (41.28%)	
Past-smoker	498 (12.89%)	692 (17.99%)	813 (20.91%)	949 (24.55%)	
Current-smoker	432 (11.18%)	725 (18.85%)	1003 (25.80%)	1321 (34.17%)	
Exercise habits					0.011
No	3167 (81.98%)	3127 (81.28%)	3211 (82.59%)	3250 (84.07%)	
Yes	696 (18.02%)	720 (18.72%)	677 (17.41%)	616 (15.93%)	
SBP (mmHg)	108.35 ± 13.01	112.12 ± 14.00	116.03 ± 14.67	121.46 ± 14.90	< 0.001
DBP (mmHg)	67.02 ± 9.20	69.83 ± 9.81	72.76 ± 10.12	76.69 ± 10.33	< 0.001
BMI (kg/m^2^)	20.45 ± 2.39	21.41 ± 2.70	22.48 ± 2.98	24.11 ± 3.14	< 0.001
ALT (IU/L)	14 (11–18)	15 (12–20)	18 (14–23)	23 (17–32)	< 0.001
AST (IU/L)	16 (13–19)	17 (14–20)	17 (14–21)	19 (16–24)	< 0.001
GGT (IU/L)	12 (10–15)	14 (11–18)	16 (12–23)	22 (16–34)	< 0.001
HDL-C (mg/dL)	65.28 ± 14.82	60.80 ± 14.82	54.30 ± 13.53	45.82 ± 11.53	< 0.001
TG (mg/dL)	34 (27–39)	54 (49–59)	79 (71–88)	136 (114–173)	< 0.001
TC (mg/dL)	181.23 ± 29.38	193.41 ± 29.90	202.15 ± 30.67	215.96 ± 33.56	< 0.001
FPG (mg/dL)	89.33 ± 6.97	92.03 ± 7.12	93.99 ± 6.93	96.52 ± 6.78	< 0.001
HbA1c (%)	5.09 ± 0.29	5.14 ± 0.31	5.19 ± 0.32	5.27 ± 0.34	< 0.001
TyG-i	7.24 ± 0.31	7.81 ± 0.14	8.22 ± 0.15	8.86 ± 0.35	< 0.001

Values are presented as n (%) or mean ± SD or median (quartile).

TyH-i: triglyceride-glycated hemoglobin index; SBP: systolic blood pressure; DBP: diastolic blood pressure; BMI: body mass index; ALT: alanine aminotransferase; AST: aspartate aminotransferase; GGT: gamma-glutamyl transferase; HDL-C: high-density lipoprotein cholesterol; TC: total cholesterol; TG: triglycerides; HbA1c: hemoglobin A1c; FPG: fasting plasma glucose; TyG-i: triglyceride-glucose index.

### The incidence rate of T2D

Over a median follow-up period of 5.39 years, a total of 373 individuals were diagnosed with T2D, resulting in a cumulative incidence of 2.41% (95% CI: 2.17–2.65) and an incidence rate of 3.99 per 1,000 person-years (Table 2). The cumulative incidence and incidence rate of T2D increased progressively across quartiles of TyH-i. Participants in the highest quartile (Q4) exhibited the highest cumulative incidence (5.79%, 95% CI: 5.06–6.53) and incidence rate (9.51 per 1,000 person-years), whereas those in the lowest quartile (Q1) had the lowest cumulative incidence (0.62%, 95% CI: 0.37–0.87) and incidence rate (1.07 per 1,000 person-years). A significant upward trend was observed across the TyH-i quartiles.

**Table 2:** Incidence rate of incident type 2 diabetes.

TyH-i	Participants (*n*)	Type 2 diabetes events (*n*)	Cumulative incidence (95% CI)(%)	Per 1,000 person-year
Total	15,464	373	2.41 (2.17–2.65)	3.99
Q1	3863	24	0.62 (0.37–0.87)	1.07
Q2	3847	40	1.04 (0.72–1.36)	1.72
Q3	3888	85	2.19 (1.73–2.65)	3.50
Q4	3866	224	5.79 (5.06–6.53)	9.51
P for trend			< 0.001	< 0.001

TyH-i: triglyceride-glycated hemoglobin index; CI: confidence interval.

**Fig. 2:**
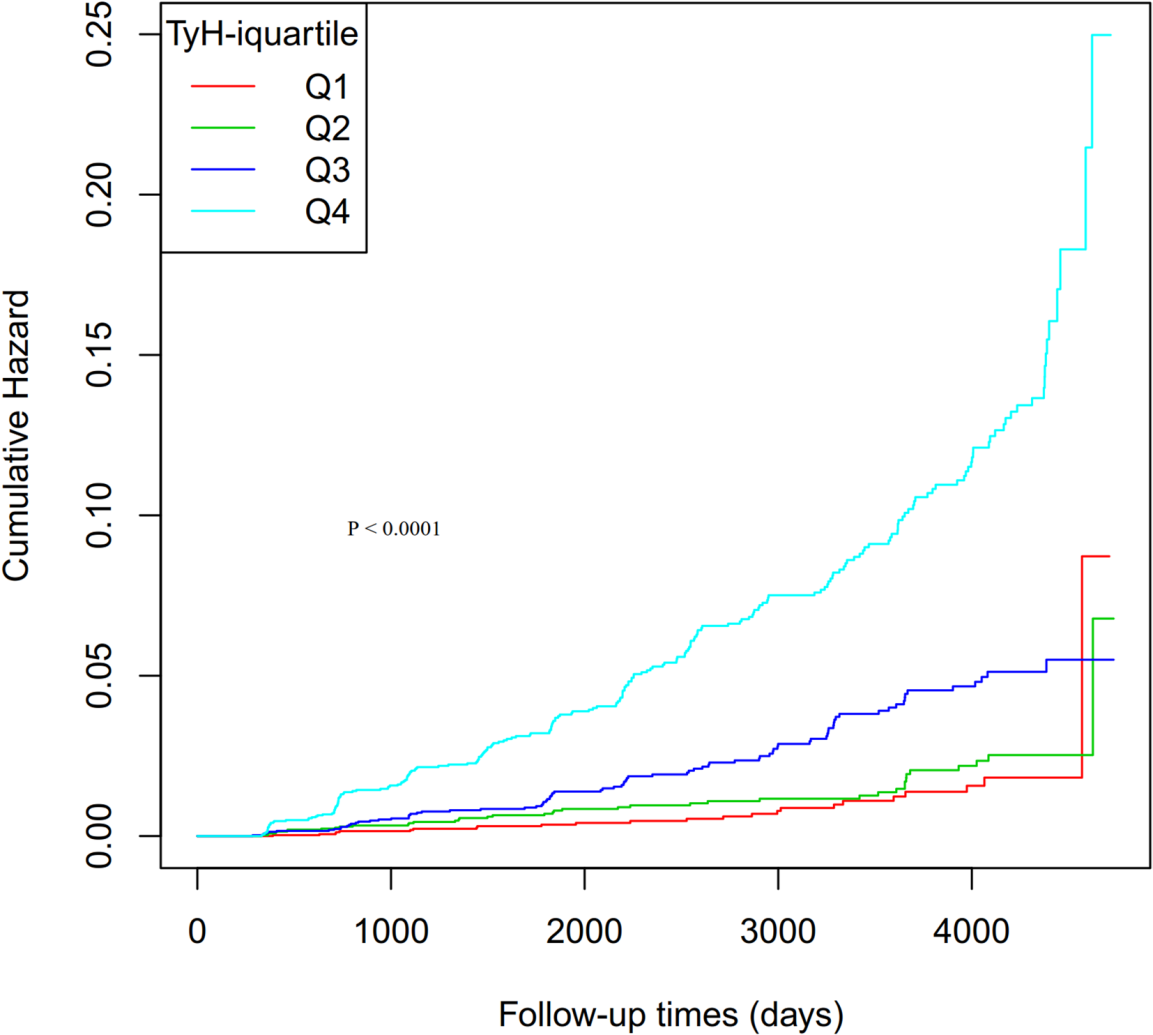
Kaplan-Meier Cumulative Hazard Curve for type 2 diabetes based on TyH-i quartiles (log-rank, *P* < 0.0001).

Additionally, Fig. 2 illustrates the Kaplan-Meier cumulative hazard curves for T2D stratified by TyH-i quartiles. The analysis revealed a clear gradient in T2D risk across the quartiles, with higher TyH-i levels associated with a significantly greater cumulative hazard of T2D (log-rank test, *P* < 0.0001). Participants in the highest TyH-i quartile (Q4) exhibited the steepest increase in T2D risk over time, while those in the lowest quartile (Q1) had the lowest cumulative hazard.

### The results of the effect of TyH-i levels on T2D

Table 3 presents the effect of TyH-i levels on T2D across three models. In the unadjusted model (Model 1), elevated TyH-i levels were positively linked to T2D (HR: 4.00, 95% CI: 3.42–4.69, *P* < 0.0001). This correlation remained robust in Model 2 (HR: 2.41, 95% CI: 1.99–2.92, *P* < 0.0001), which adjusted for alcoholic intake, sex, exercise habits, age, BMI, smoking status, and SBP. In Model 3, which additionally accounted for ALT, TC, AST, HDL-C, GGT, and FPG, TyH-i remained independently associated with T2D risk (HR: 1.55, 95% CI: 1.22–1.97, *P* = 0.0003). Notably, in Model 3, individuals in the Q4 group exhibited a 21% higher risk of T2D (HR: (HR: 1.21, 95% CI: 0.74–2.00) than those in the Q1 group.

**Table 3:** Relationship between TyH-i and incident type 2 diabetes in different models.

Variable	Model 1 (HR, 95%CI, *P*)	Model 2 (HR, 95% CI, *P*)	Model 3 (HR, 95% CI, *P*)
TyH-i	4.00 (3.42, 4.69) < 0.0001	2.41 (1.99, 2.92) < 0.0001	1.55 (1.22, 1.97) 0.0003
TyH-i (quartile)			
Q1	Ref	Ref	Ref
Q2	1.53 (0.92, 2.54) 0.0990	1.06 (0.64, 1.76) 0.8267	0.83 (0.50, 1.40) 0.4911
Q3	3.09 (1.96, 4.86) < 0.0001	1.48 (0.92, 2.36) 0.1027	0.88 (0.54, 1.43) 0.6126
Q4	8.48 (5.56, 12.92) < 0.0001	2.74 (1.74, 4.31) < 0.0001	1.21 (0.74, 2.00) 0.4474
P for trend	< 0.0001	< 0.0001	0.0834

Model 1: we did not adjust for any covariates.

Model 2: we adjusted for sex, age, BMI, alcoholic intake, smoking status, exercise habits, and SBP.

Model 3: we adjusted for sex, age, BMI, alcoholic intake, smoking status, exercise habits, SBP, ALT, AST, GGT, TC, HDL-C, and FPG.

HR: hazard ratio; CI: confidence interval; Ref: Reference; TyH-i: triglyceride-glycated hemoglobin index.

### Sensitive analysis

Sensitivity analyses were conducted to confirm the robustness of the findings (Table 4). In participants with BMI < 25 kg/m^2^ (Model 4), TyH-i remained significantly linked to T2D risk (HR: 1.57, 95% CI: 1.14–2.15, *P* = 0.0053). Similarly, in participants younger than 60 years (Model 5), the association persisted (HR: 1.59, 95% CI: 1.24–2.04, *P* = 0.0003).

**Table 4:** Relationship between TyH-i and incident type 2 diabetes in different sensitivity analyses.

Variable	Model 4 (HR, 95%CI, *P*)	Model 5 (HR, 95% CI, *P*)
TyH-i	1.57 (1.14, 2.15) 0.0053	1.59 (1.24, 2.04) 0.0003
TyH-i (quartile)		
Q1	Ref	Ref
Q2	0.99 (0.55, 1.78) 0.9625	0.79 (0.46, 1.35) 0.3853
Q3	0.96 (0.53, 1.71) 0.8819	0.88 (0.53, 1.47) 0.6301
Q4	1.48 (0.80, 2.73) 0.2116	1.20 (0.71, 2.04) 0.4902
P for trend	0.0877	0.0844

Model 4 was a sensitivity analysis in participants with BMI < 25 kg/m^2^. We adjusted for sex, age, BMI, alcoholic intake, smoking status, exercise habits, SBP, ALT, AST, GGT, TC, HDL-C, and FPG.

Model 5 was a sensitivity analysis in individuals aged < 60 years. We adjusted for sex, age, BMI, alcoholic intake, smoking status, exercise habits, SBP, ALT, AST, GGT, TC, HDL-C, and FPG.

HR: hazard ratio; CI: confidence interval; Ref: Reference; TyH-i: triglyceride-glycated hemoglobin index.

### Nonlinear relationship between TyH-i and T2D

**Fig. 3:**
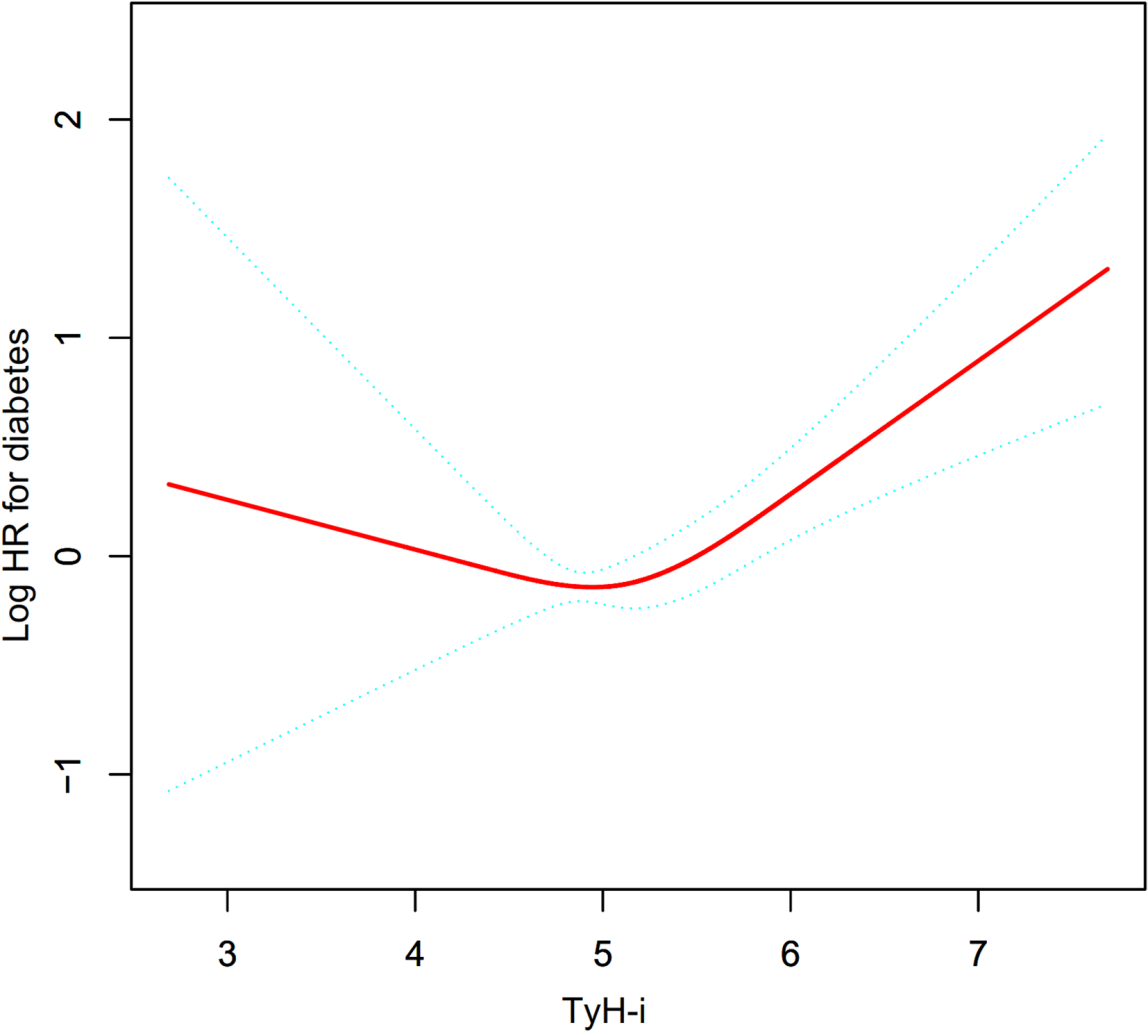
The nonlinear relationship between TyH-i and incident type 2 diabetes. The nonlinear relationship was observed after adjusting for multiple confounding factors, including sex, age, BMI, alcoholic intake, smoking status, exercise habits, SBP, ALT, AST, GGT, TC, HDL-C, and FPG.

Table 5 and Fig. 3 illustrate a nonlinear association between TyH-i and T2D. An inflection point for TyH-i was identified at 4.92. Below this threshold, no significant association was observed between TyH-i and T2D (HR: 0.61, 95% CI: 0.28–1.34, *P* = 0.2161). However, above the inflection point, TyH-i was strongly and positively linked to an elevated risk of T2D (HR: 1.73, 95% CI: 1.35–2.23, *P* < 0.0001).

**Table 5:** The result of the two-piecewise Cox proportional hazards regression model.

Incident type 2 diabetes	HR (95%CI)	*P*
Fitting model by two-piecewise Cox proportional hazards regression		
The inflection point of TyH-i	4.92	
≤ 4.92	0.61 (0.28, 1.34)	0.2161
> 4.92	1.73 (1.35, 2.23)	< 0.0001
P for the log-likelihood ratio test	0.026	

We adjusted sex, age, BMI, alcoholic intake, smoking status, exercise habits, SBP, ALT, AST, GGT, TC, HDL-C, and FPG.

HR: hazard ratios; CI: confidence; TyH-i: triglyceride-glycated hemoglobin index.

### Subgroup analysis

The effect of TyH-i on T2D risk was further analyzed in prespecified and exploratory subgroups (Table 6). No significant interactions were observed between TyH-i and subgroup characteristics, including age, sex, alcohol intake, smoking status, exercise habits, and BMI (all P for interaction > 0.05).

**Table 6:** Effect size of TyH-i on type 2 diabetes in prespecified and exploratory subgroups.

Characteristic	No of participants	HR (95%CI)	*P* value	*P* for interaction
Age(years)				0.8356
< 45	8872	1.69 (1.18, 2.42) 0.0040		
45–60	5880	1.53 (1.08, 2.15) 0.0157		
≥ 60	712	1.69 (0.79, 3.61) 0.1721		
Sex				0.1845
Female	7034	1.97 (1.29, 3.03)	0.0018	
Male	8430	1.47 (1.14, 1.89)	0.0028	
Alcoholic intake (g/wk)				0.1115
= 0	4735	2.15 (1.34, 3.46)	0.0015	
> 0	10,729	1.39 (1.06, 1.82)	0.0173	
Smoking status				0.6772
Never-smoker	9031	1.59 (1.08, 2.34)	0.0188	
Past-smoker	2952	1.54 (0.95, 2.47)	0.0785	
Current-smoker	3481	1.42 (0.99, 2.05)	0.0583	
Exercise habits				0.4588
No	12,755	1.61 (1.25, 2.06)	0.0002	
Yes	2709	1.31 (0.77, 2.22)	0.3197	
BMI (kg/m^2^)				0.5648
< 25	12,940	1.64 (1.21, 2.24)	0.0017	
25–30	2258	1.46 (0.98, 2.16)	0.0611	
≥ 30	266	1.35 (0.55, 3.27)	0.5116	

Note 1: The above model adjusted for we adjusted for sex, age, BMI, alcoholic intake, smoking status, exercise habits, SBP, ALT, AST, GGT, TC, HDL-C, and FPG.

Note 2: The model is not adjusted for the stratification variable in each case.

### Diagnostic performance of TyH-i in identifying T2D

**Fig. 4:**
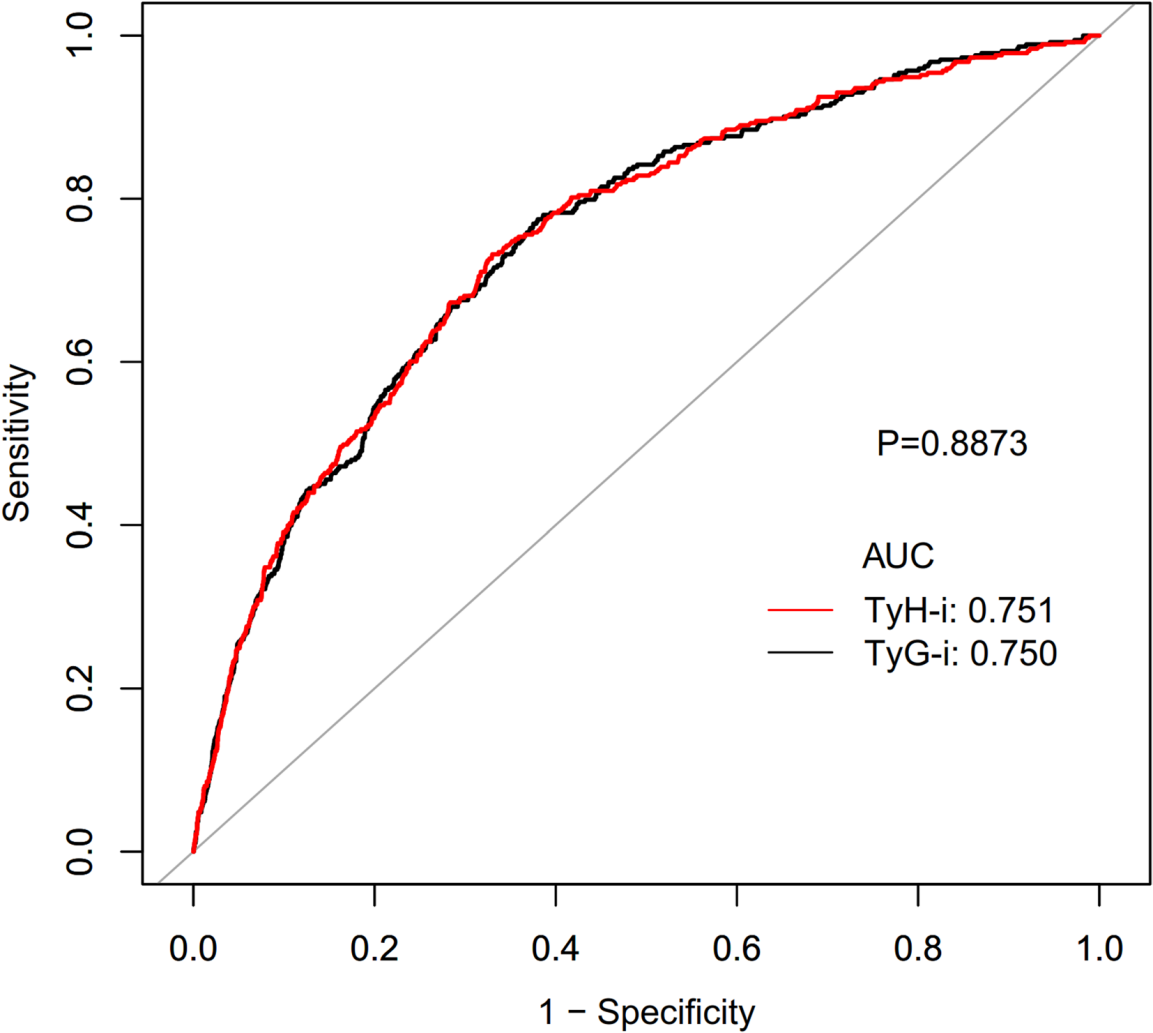
The TyH-i for predicting type 2 diabetes in all participants by ROC analyses.

The diagnostic performance of TyH-i in identifying T2D was assessed through ROC curve analysis (Table 7; Fig. 4). The AUC for TyH-i was 0.751 (95% CI: 0.726–0.776), demonstrating good discriminatory ability. The optimal threshold for TyH-i was identified as 5.380, corresponding to a specificity of 67.0% and a sensitivity of 73.2%, with a resulting Youden Index of 0.402. The AUC for TyG-i was 0.750 (95% CI: 0.726–0.775). The optimal threshold for TyG-i was determined to be 8.196, with a specificity of 62.0% and a sensitivity of 77.5%, resulting in a Youden Index of 0.395. TyH-i and TyG-i possess comparable discriminatory ability in predicting incident T2D (AUC: 0.751 vs. 0.750, *P* = 0.8873). Moreover, TyH-i yielded lower AIC and BIC values than TyG-i (AIC: 3211.60 vs. 3211.91; BIC: 3226.89 vs. 3227.20) (Table 8). Calibration of the TyH-i and TyG-i models was assessed using calibration plots generated from 1,000 bootstrap resamples and the Hosmer-Lemeshow goodness-of-fit test (Fig. S1 and S2). The Hosmer-Lemeshow test yielded non-significant P-values (*P* = 0.1873 for TyH-i and *P* = 0.2164 for TyG-i). These findings demonstrate that both the TyH-i and TyG-i models exhibit excellent calibration and can reliably estimate the risk of T2D. Furthermore, external validation was conducted in a cohort of 8,304 Chinese participants. The AUC was 0.608 (95%CI: 0.591–0.626) for TyH-i and 0.605 (95%CI: 0.587–0.623) for TyG-i (Table S2 and Fig. S3). At the optimal cutoff, the specificity and sensitivity for TyH-i were 57.3% and 60.5%, respectively, while for TyG-i, the corresponding values were 48.0% and 67.8%.

**Table 7:** Areas under the receiver operating characteristic curves for each evaluated parameter in identifying type 2 diabetes.

Test	AUC	95%CI	Best threshold	Specificity	Sensitivity	Youden index	AIC	BIC
TyH-i	0.751	0.726–0.776	5.380	0.670	0.732	0.402	3211.60	3226.89
TyG-i	0.750	0.726–0.775	8.196	0.620	0.775	0.395	3211.91	3227.20

TyH-i: triglyceride-glycated hemoglobin index; TyG-i: triglyceride-glucose index.

**Table 8:** Comparative analysis of TyH-i and TyG-i for type 2 diabetes risk prediction: evaluation using net reclassification improvement (NRI) and integrated discrimination improvement (IDI).

Test	Estimate (95%CI)	Standard Error	*P*-value
NRI	0.0073 (−0.0136–0.0282)	0.0107	0.4954
IDI	0.0073 (0.0107–0.6809)	0.0107	0.4960

## Discussion

This retrospective study investigated the correlation between TyH-i and T2D in a population of 15,464 participants without T2D at baseline. Our findings revealed that higher TyH-i levels remained significantly linked to a higher risk of T2D, even after adjusting for confounders. TyH-i and the TyG-i demonstrated comparable discriminatory abilities. Sensitivity and subgroup analyses consistently confirmed the robust association between TyH-i and T2D risk. These findings suggest that TyH-i could be a valuable and practical indicator for identifying individuals at elevated risk of developing T2D.

Insulin resistance is a fundamental feature in the pathogenesis of T2D and plays a critical role in the progression from normoglycemia to prediabetes and, ultimately, to diabetes^18^. TG and HbA1c, the two components of the TyH-i, are strongly linked to insulin resistance and metabolic dysfunction^19–22^. Previous studies have extensively investigated the relationships between TG, HbA1c, and T2D. For instance, a retrospective cohort analysis of over 30,000 participants demonstrated, through multivariate analyses, that elevated triglyceride levels within the normal range were linked to a higher risk of diabetes^23^. Similarly, a cross-sectional study of 20,108 diabetes patients found a J-shaped correlation between triglyceride levels and poor glycemic control after adjusting for confounding factors^24^. Furthermore, a prospective cohort study of 11,092 non-diabetic adults revealed that higher HbA1c levels, even within the nondiabetic range, were significantly linked to a higher risk of developing cardiovascular disease and diabetes, as shown by multifactorial outcome analyses^25^. Additionally, a systematic review encompassing 16 studies with a total of 44,203 participants found that HbA1c levels were strongly predictive of future diabetes, with individuals in the prediabetic range (5.7–6.4%) exhibiting a significantly higher risk of developing diabetes compared to those with lower HbA1c levels^26^. These findings underscore the critical roles of TG and HbA1c in metabolic dysfunction and their potential utility in predicting diabetes risk. Although there is no prior data on the association between TyH-i and the likelihood of developing T2D, an increase in TyH-i reflects either an elevation in triglyceride levels or an increase in HbA1c. Consequently, our results support the existing evidence that TyH-i is independently linked to T2D risk. Moreover, our sensitivity analysis demonstrated that the association between TyH-i and T2D risk remained robust in individuals with age < 60 years or BMI < 25 kg/m^2^. A higher TyH-i during the follow-up period indicates an elevated risk of T2D, underscoring the importance of early lifestyle interventions to proactively reduce the incidence of T2D.

A key finding of our study was the identification of a significant nonlinear relationship between TyH-i and T2D risk. We identified an inflection point at a TyH-i value (TyH-i = 4.92) through two-piecewise Cox proportional hazards regression analysis. Below this threshold, the correlation between TyH-i and T2D was not statistically significant. However, above this threshold, each unit increase in TyH-i was linked to a 73% higher risk of T2D. These results suggest that elevated TyH-i could be a valuable indicator for identifying individuals at an elevated risk of hyperglycemia, highlighting the importance of early lifestyle interventions to improve long-term health outcomes.

The observed association between TyH-i and T2D risk can be attributed to several underlying biological mechanisms. TyH-i represents the combined effects of triglycerides and glycated hemoglobin, both of which are strongly associated with insulin resistance and glucose dysregulation. Elevated triglyceride levels, a hallmark of metabolic syndrome, are linked to impaired insulin signaling and pancreatic β-cell dysfunction, both of which play critical roles in the pathogenesis of diabetes^27,28^. Similarly, glycated hemoglobin reflects chronic hyperglycemia and serves as a well-established predictor of T2D^13,15^.

The current study possesses several notable strengths. First, it is based on a large cohort with a long follow-up period, allowing for a robust assessment of the correlation between TyH-i and T2D risk. Second, the use of multiple statistical models and sensitivity analyses enhances the reliability and generalizability of our findings. Third, the comparison of TyH-i with TyG-i provides valuable insights into the relative utility of these indices in predicting T2D.

However, several limitations should be acknowledged. First, although we adjusted for a series of confounders, residual confounding could not be entirely excluded. Second, the study population was performed in a Japanese population, which might limit the generalisability of our results to other populations. Third, our study is a retrospective cohort study, and further experimental or interventional studies are required to confirm a causal correlation between TyH-i and T2D risk. Fourth, all participants who were taking any medication at baseline were excluded from our study. We acknowledge that this exclusion criterion may have impacted the representativeness of our sample. Unfortunately, the DATADRYAD database, which was the source of our data, does not provide information regarding the characteristics of the excluded population. In future research, we aim to validate the association between TyH-i and T2D in more diverse populations, including individuals who are taking medications at baseline.

## Conclusion

Our study demonstrates that TyH-i is independently associated with an increased risk of T2D and exhibits a nonlinear relationship with T2D risk, with a steeper increase observed at higher TyH-i levels. These findings highlight the potential of TyH-i as a valuable and practical marker for T2D risk stratification.

## Electronic supplementary material

Below is the link to the electronic supplementary material.

Supplementary Material 1

Supplementary Material 2

Supplementary Material 3

Supplementary Material 4

## Data Availability

The raw data can be downloaded from the ‘DATADRYAD’ database (www.Datadryad.org). Dryad Digital Repository. https://datadryad.org/stash/dataset/doi:10.5061%2Fdryad.8q0p192.

## Abbreviations

T2D: Type 2 diabetes
TyH-i: Triglyceride-glycated hemoglobin index
TyG-i: Triglyceride-glucose index
HbA1c: Glycated hemoglobin
TG: Triglyceride
BMI: Body mass index
SBP: Systolic blood pressure
DBP: Diastolic blood pressure
ALT: Alanine aminotransferase
AST: Aspartate aminotransferase
GGT: Gamma-glutamyl transferase
HDL-C: High-density lipoprotein cholesterol
TC: Total cholesterol
FPG: Fasting plasma glucose
IQR: Interquartile ranges
SD: Standard deviations
HR: Hazard ratio
CI: Confidence interval
