# Supplementary material for: Triglyceride-glycated hemoglobin index as a superior predictor of type 2 diabetes risk in a large-scale retrospective cohort study
Source: Sci Rep. 2025 Jul 1;15:20898. doi: 10.1038/s41598-025-05786-4 (PMC12216214; doi:10.1038/s41598-025-05786-4)
Supplement: Supplementary file 1 — Supplementary Material 1 [file 41598_2025_5786_MOESM1_ESM.docx]

**Table S1** The results of the collinearity screening

|  | Step 1 | Step 2 |
| --- | --- | --- |
| **Sex** | 1.9 | 1.9 |
| **Age (years)** | 1.2 | 1.2 |
| **Alcoholic intake (g/wk)** | 1.3 | 1.3 |
| **Smoking status** | 1.4 | 1.4 |
| **Exercise habits** | 1.0 | 1.0 |
| **SBP (mmHg)** | 5.6 | 1.4 |
| **DBP (mmHg)** | 5.7 | NA |
| **BMI (kg/m^2^)** | 1.7 | 1.7 |
| **ALT (IU/L)** | 3.9 | 3.9 |
| **AST (IU/L)** | 3.2 | 3.2 |
| **GGT (IU/L)** | 1.5 | 1.5 |
| **HDL-C (mg/dL)** | 1.5 | 1.5 |
| **TC (mg/dL)** | 1.3 | 1.3 |
| **FPG (mg/dL)** | 1.3 | 1.3 |

**Table S2** Areas under the receiver operating characteristic curves for each evaluated parameter in identifying type 2 diabetes in middle-aged and older adults from the China health and retirement longitudinal study

| Test | AUC | 95%CI | Best threshold | Specificity | Sensitivity | Youden Index |
| --- | --- | --- | --- | --- | --- | --- |
| TyH-i | 0.608 | 0.591-0.626 | 5.504 | 0.573 | 0.605 | 0.178 |
| TyG-i | 0.605 | 0.587-0.623 | 8.590 | 0.480 | 0.678 | 0.158 |

TyH-i: triglyceride-glycated hemoglobin index; TyG-i: triglyceride-glucose index

**Figure S1** Calibration and comparative predictive performance of TyH-i for type 2 diabetes

**Figure S2** Calibration and comparative predictive performance of TyG-i for type 2 diabetes

**Figure S3** The TyH-i for predicting type 2 diabetes by ROC analyses in middle-aged and older adults from the China health and retirement longitudinal study.
