# Supplementary figures and images for: Triglyceride-glycated hemoglobin index as a superior predictor of type 2 diabetes risk in a large-scale retrospective cohort study
Source: Sci Rep. 2025 Jul 1;15:20898. doi: 10.1038/s41598-025-05786-4 (PMC12216214; doi:10.1038/s41598-025-05786-4)

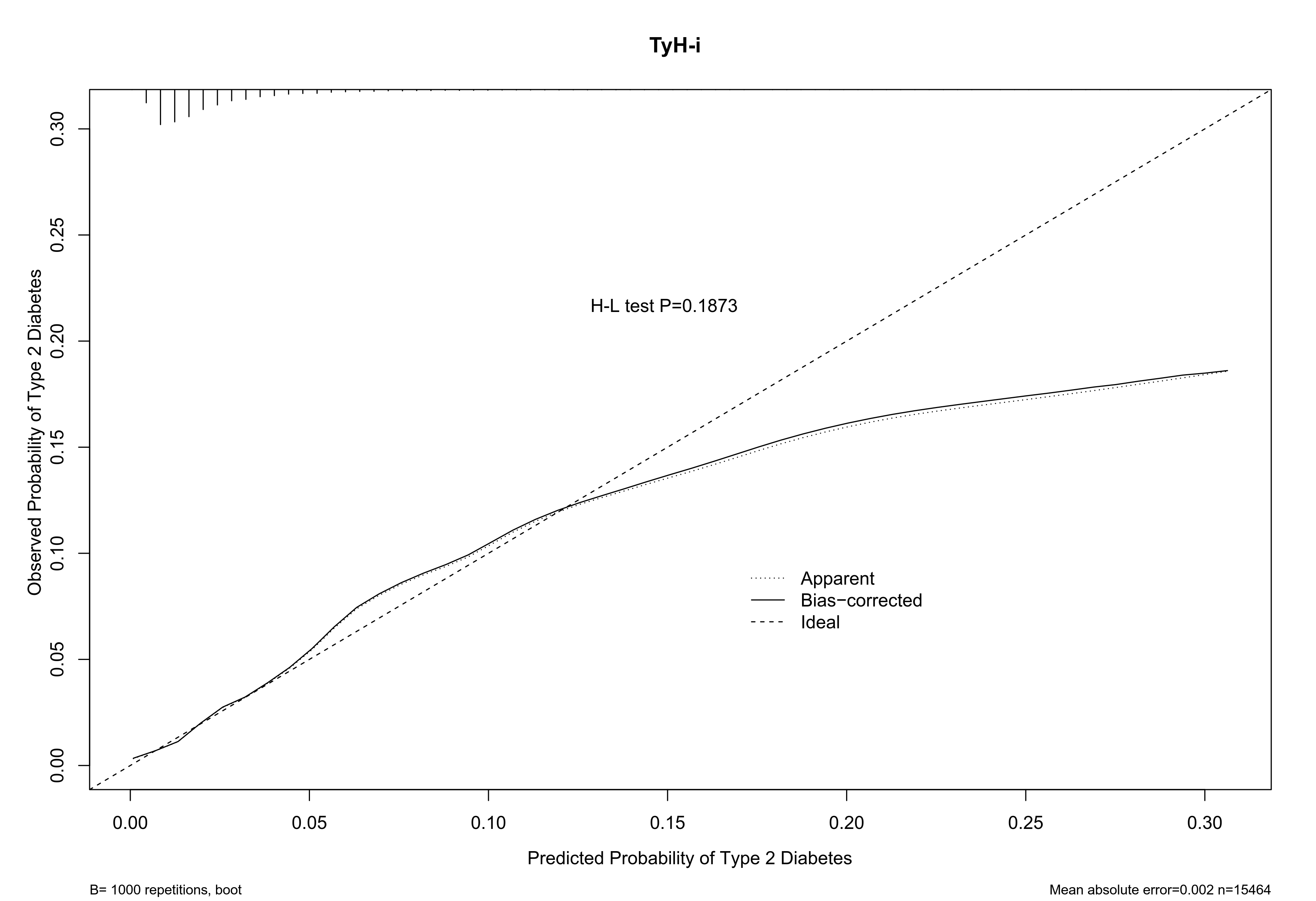

Supplement: Supplementary file 2 — Supplementary Material 2 [file 41598_2025_5786_MOESM2_ESM.tif]

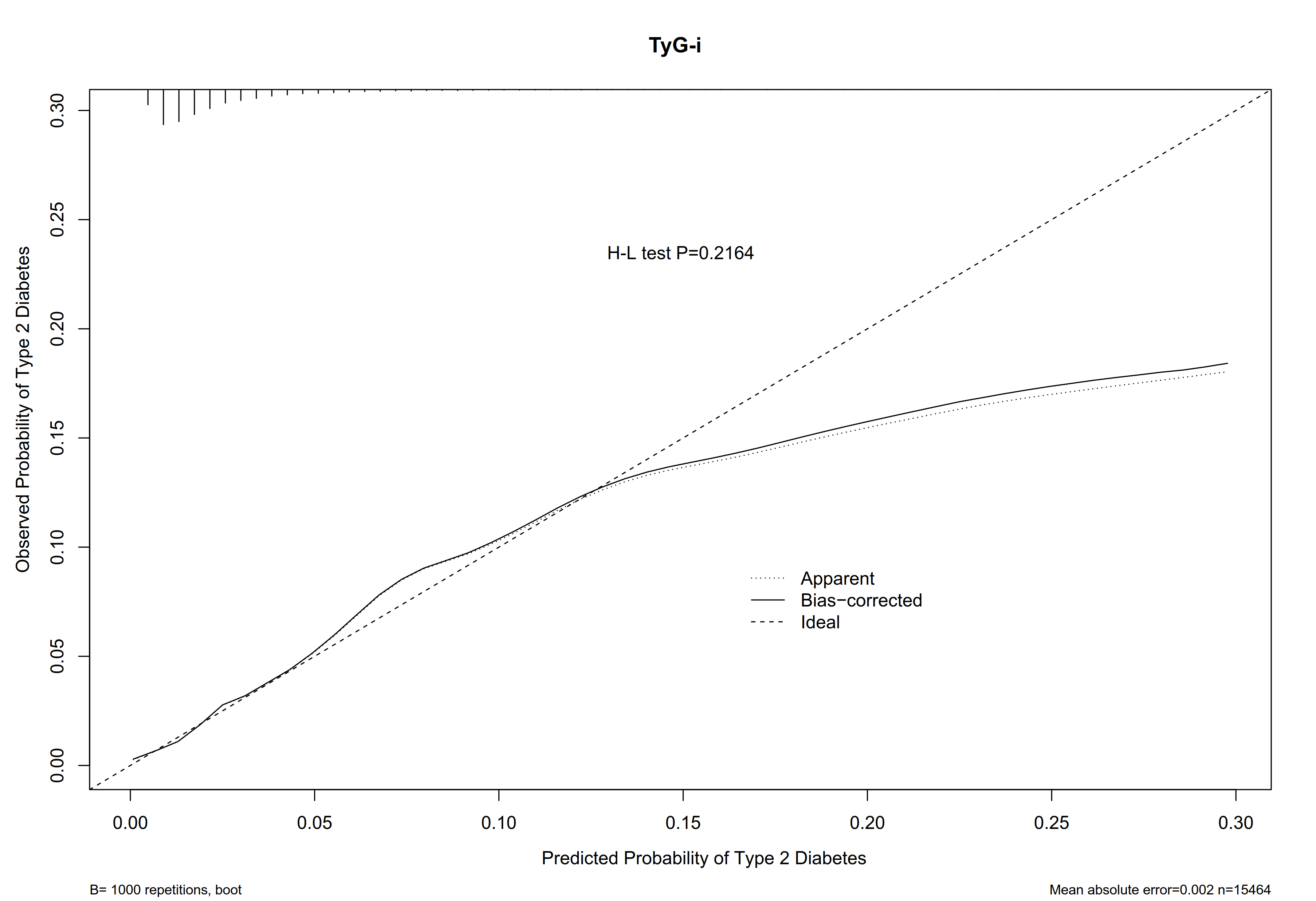

Supplement: Supplementary file 3 — Supplementary Material 3 [file 41598_2025_5786_MOESM3_ESM.tif]

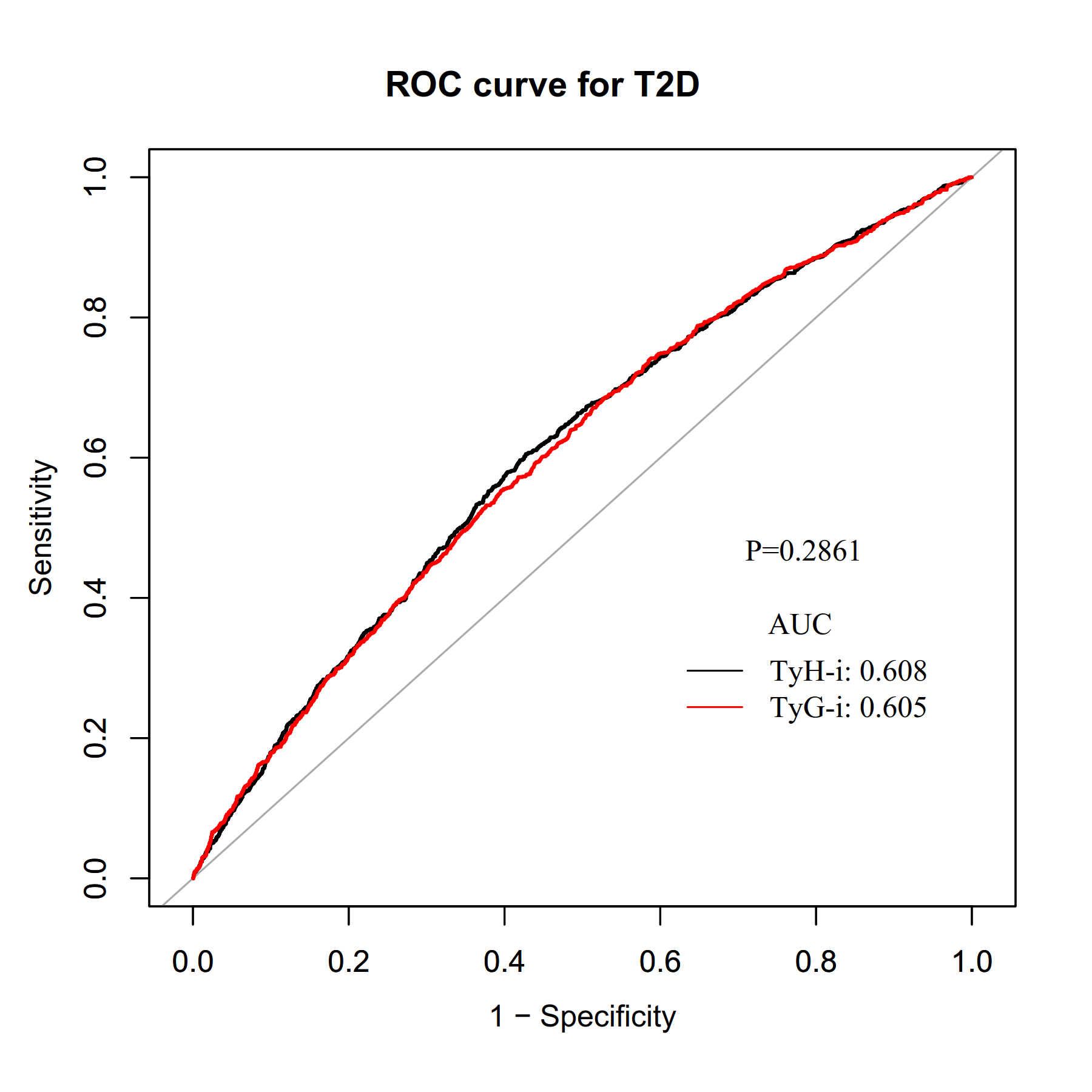

Supplement: Supplementary file 4 — Supplementary Material 4 [file 41598_2025_5786_MOESM4_ESM.tif]
